# Highly heterogeneous diazotroph communities in the Kuroshio Current and the Tokara Strait, Japan

**DOI:** 10.1371/journal.pone.0186875

**Published:** 2017-10-23

**Authors:** Shunyan Cheung, Koji Suzuki, Hiroaki Saito, Yu Umezawa, Xiaomin Xia, Hongbin Liu

**Affiliations:** 1 Division of Life Science, The Hong Kong University of Science and Technology, Hong Kong, China; 2 Faculty of Environmental Earth Science, Hokkaido University, Sapporo, Japan; 3 Atmosphere and Ocean Research Institute, The University of Tokyo, Tokyo, Japan; 4 Faculty of Fisheries, Nagasaki University, Nagasaki, Japan; University of Connecticut, UNITED STATES

## Abstract

In this study, we used 454-pyrosequencing to report the highly diverse diazotroph communities in the Kuroshio and its adjacent waters along a transect across the Tokara Strait, Japan. Terrestrial input from the islands resulted in a highly heterogeneous diazotroph community within a relatively small geographic region, which was presumably caused by the remarkably different responses of UCYN-A2, UCYN-C and *Trichodesmium* to the steep environmental gradient. On the other hand, most major cyanobacterial OTUs found in this study were also detected in an unpublished dataset from the upstream Kuroshio, which suggests transportation of diazotrophs by the Kuroshio in large geographic scale. A significant amount of UCYN-C was found in the Kuroshio and offshore stations, suggesting the importance of this potentially overlooked group in the western North Pacific Ocean (WNPO). Moreover, a novel sublineage of UCYN-B was defined, which was predominant in an oligotrophic water sample; and it was also found to be widely distributed in oceanic waters. In addition, the apparent increase in relative abundance of UCYN-A2 from offshore to near-shore water provides evidence for the earlier and under-debating view that UCYN-A2 prefers coastal conditions. Our report provides new knowledge for understanding the phylogeny and ecology of unicellular cyanobacterial diazotrophs in WNPO.

## Introduction

Biological nitrogen fixation is an important source of new nitrogen input in the subtropical and tropical oceans [1–3], where bioavailable nitrogen is usually a major limiting factor for primary production. Biological nitrogen fixation is carried out by a group of prokaryotes, called diazotrophs. *Trichodesmium* was initially believed to be the major diazotroph in the oceans [4]. However, with the development of molecular techniques, and the *nifH* gene (which encodes a nitrogenase subunit), becoming a commonly-used phylogenetic biomarker of the diazotrophs [5], a diverse range of microbes has since been discovered. Thus, more recent studies indicate that the contribution of unicellular cyanobacterial diazotrophs to nitrogen fixation could be equal to (or even higher than) that of *Trichodesmium* [6, 7].

The marine unicellular cyanobacterial diazotrophs are divided into 3 distinct lineages namely, UCYN-A, UCYN-B and UCYN-C, and these have been detected in diverse environments [8]. Several strains of UCYN-B, including the widely-distributed strain *Crocosphaera watsonii* WH8501 [9], have been isolated and relatively well studied. In contrast, UCYN-A and UCYN-C are less well understood; likely due to the fact that UCYN-A is still uncultivated and there is just one cultured strain of UCYN-C (*Cyanothece* sp. TW3) [10]. However, when comparing UCYN-A with UCYN-C, the former has been studied in more detail because it can be detected in high abundance at higher latitudes and in deeper water than other cyanobacterial diazotrophs [11]. Indeed, it has been shown that the UCYN-A group lacks both the CO_2_-fixing enzyme, RubisCO, and the oxygen-evolving photosystem II [12]. It is also known to form a symbiotic relationship with picoeukaryotic prymnesiophtyes [13]. In addition, different sublineages of UCYN-A have been identified in the oceans, and so additional physiological information about this linage is now available [14, 15].

Kuroshio is a western boundary current in the North Pacific Ocean. It originates from the North Equatorial Current, enters the East China Sea and flows out through the Tokara Strait to the Pacific Ocean [16]. The Kuroshio Current (KC) is also generally characterized as having oligotrophic conditions and a deep nitracline. It was suggested that such conditions are advantageous for the survival of diazotrophs over non-diazotrophs [17]. In the oligotrophic surface water of the KC, biological nitrogen fixation was reported to be strong (232 ± 54.8 μmol N m^−2^ d^−1^) [18] and was estimated to contribute up to 25% of the new production [19]. Early studies reported that the abundance of *Trichodesmium* was higher in the KC than in the neighboring East China Sea, and they were recognized as being an important diazotroph in this area [20]. However, a more recent study conducted in the upstream region of the KC reported that the filamental portion of nitrogen fixation was dominant during the warm season, whereas the unicellular portion contributed to ~70% of the nitrogen fixation that occurs during the cold season [21]. Thus far, however, the major unicellular diazotrophs in the KC have not been identified. Although the gene copy numbers of UCYN-A1 and UCYN-B have been quantified in the upstream region of KC, these two clades cannot represent the entire community of unicellular diazotrophs. This is because the unicellular diazotrophs are a big family, comprising different clades of UCYN-A, B, and C, as well as some proteobacterial phylotypes, which have all been suggested to be significant diazotrophs in the marine ecosystem [8, 14, 22]. Furthermore, the 18S rRNA gene sequence of *Braarudosphaera* sp., a symbiotic host of UCYN-A2, has also been detected in the coastal waters of Japan [14, 23], which implies that diverse unicellular diazotrophs might inhabit the KC and the adjacent waters of the Tokara Strait. Nevertheless, since the discovery of the sublineages of the UCYN-A, the detailed distributional information about these sublineages has been missing in the oceanic water of the western North Pacific Oceans [24, 25].

Therefore, in this study we used 454-pyrosequecing, deep sequencing technique to analyze the diazotroph community and active diazotrophs in the KC (oligotrophic water in western North Pacific Ocean). Besides that, we choose to study the KC in a transect along the Tokara Strait south of Kyushu Island, Japan, where steep environmental gradients are expected. This can provide more understandings about the responses of different diazotrophic phylotypes to the highly variable environmental conditions of the Ocean.

## Methods

### Environmental conditions and sample collection

**Fig 1:**
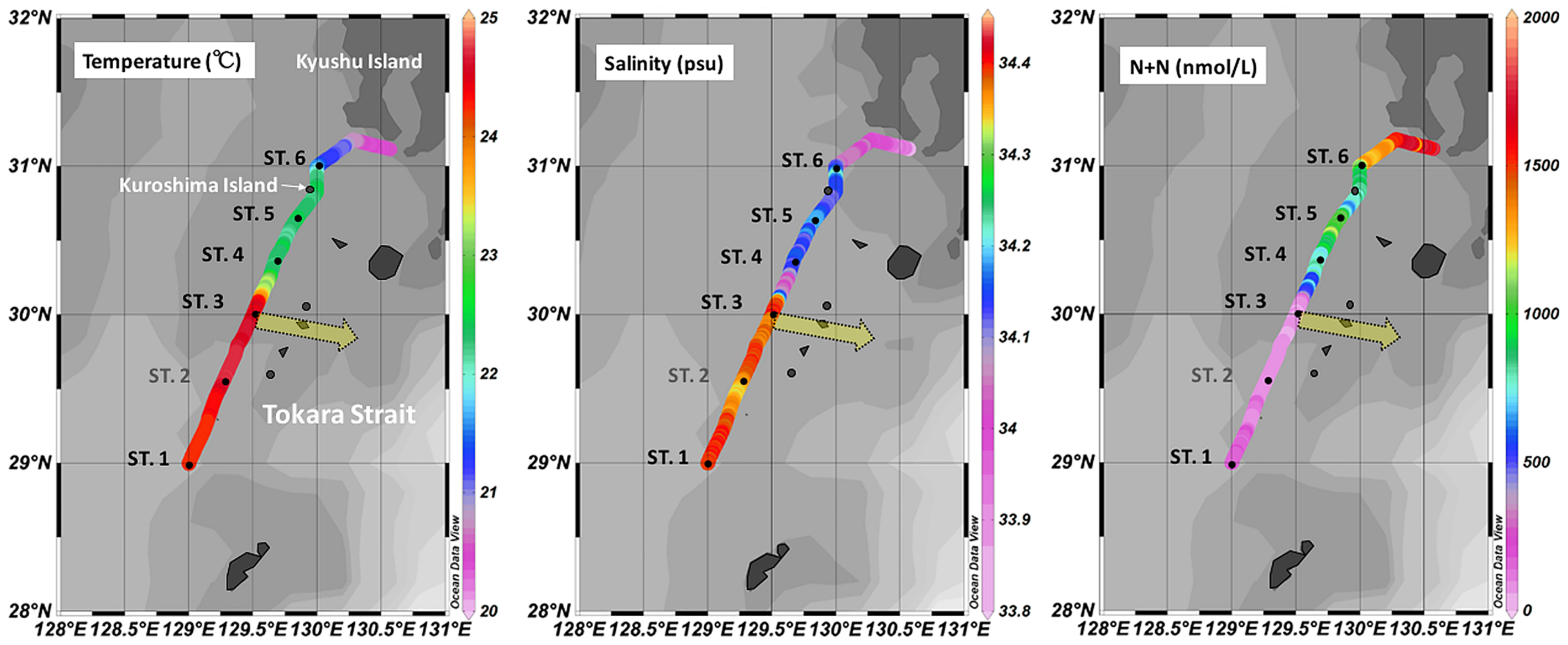
Map and surface hydrographic features of the stations. The yellow arrow represents the location and direction of the Kuroshio mainstream.

This study was conducted at 5 stations (ST.1, 3, 4, 5 and 6) in the Tokara Strait (29°0΄N—31°0΄N, 129°0΄E—130°0΄E) on board the R/V *Tansei Maru* (JAMSTEC; Table 1, Fig 1 and S1 Fig) during November, 2012. Basic hydrographic data (i.e., salinity, temperature, depth and dissolved oxygen concentration) and water samples were collected using a conductivity-temperature-depth (CTD) rosette system, with Sea Bird oxygen sensors (Sea Bird Electronics Inc., Bellevue, WA, USA) and attached Niskin bottles. In addition to the basic hydrographic data, concentrations of ammonium (NH_4_^+^), nitrite (NO_2_^-^), nitrate (NO_3_^-^), phosphate (PO_4_^3-^), and silicate (SiO_2_) were determined with a segmented flow auto-analyzer; and chlorophyll-a (*Chl a*) was measured by fluorometry [26]. For the stable isotope analysis of N of suspended materials, water samples were filtered through pre-combusted GF/F filters, and the filters were kept frozen at -30°C until analysis. To remove carbonates, the filters were exposed to HCl fumes for 24 hours, and then dried. The N isotopic compositions (δ^15^N) were determined by means of an elemental analyzer (ANCA-GSL) interfaced with an isotope ratio mass spectrometer (Hydra 20–20, Sercon Ltd., Crewe, Cheshire, UK). The surface current data were collected using a ship-mounted acoustic Doppler current profiler (ADCP) (Ocean Surveyor, Teledyne RD Instruments) during the expedition. For molecular analysis, 6–8 L seawater samples were collected from the surface layer (i.e., a depth of 5 m) and the deep chlorophyll maximum (DCM) layer at each station (Table 1), after which they were filtered onto 0.2 μm Supor^®^ PES filter membranes (47 mm, Pall Corp., Port Washington, NY, USA) under low vacuum pressure. All the molecular samples were then flash frozen and stored at -80°C until they were required for DNA and RNA extraction.

**Table 1:** Basic information about the DNA samples used in this study.

Station	Lat. (°N)	Long. (°E)	Depth (m)	Sample Name	No. of sequences	Coverage
ST.1	29.00	129.00	5	ST.1-S	3,598	0.999
			60	ST.1-C	3,661	0.998
ST.3	30.00	129.50	5	ST.3-S	3,629	0.996
			5	ST.3-SR	3,844	0.997
			60	ST.3-C	3,597	0.997
			60	ST.3-CR	3,833	0.997
ST.4	30.33	129.67	5	ST.4-S	3,744	0.998
			70	ST.4-C	3,758	0.997
ST.5	30.67	129.83	5	ST.5-S	3,641	0.999
			20	ST.5-C	3,780	0.998
ST.6	31.00	130.00	5	ST.6-S	427	0.998
			75	ST.6-C	3,772	0.999

The sequence numbers, and coverage are also listed. DCM means that samples were collected from the deep chlorophyll maximum layer. The samples collected from the surface water were labeled with “S” in their sample names, while those collected from deep chlorophyll maximum layer were labeled with “C” and the cDNA samples were labeled with “R”.

### DNA and RNA extraction, and cDNA synthesis

Genomic DNA and RNA was extracted from the 0.2 μm membrane filters using the TRIzol plus Genomic DNA mini kit and RNA Purification mini kit (Invitrogen, Thermo Fisher Scientific Corp., Carlsbad, CA, USA). All the DNA samples were eluted in 60 μL ultrapure water (Invitrogen, Thermo Fisher Scientific Corp.), and stored at -20°C until further analysis. The RNA samples were eluted in 60 μL RNase-free water (Invitrogen, Thermo Fisher Scientific Corp.) and stored at -80°C until further analysis.

For cDNA synthesis, the extracted RNA was first treated with DNase I to remove any contaminating DNA, after which it was reverse-transcribed with the SuperScript III First Strand cDNA Synthesis kit (Invitrogen, Thermo Fisher Scientific Corp.). Each reaction contained 8 μL DNase treated RNA, 0.5 μM random hexamers, 1×RT buffer, 5 mM magnesium chloride (MgCl_2_), 0.5 mM deoxynucleotide triphosphate (dNTPs), 10 mM dithiothreitol, 1 unit RNaseOUT^™^ and 0.5 unit SuperScript III reverse-transcriptase. The reaction conditions were 25°C for 10 min; 50°C for 50 min and 85°C for 5 min. The cDNA synthesized was then incubated with 1 unit RNase H at 37°C for 20 min, after which the cDNA samples were stored at -20°C until required for further analysis.

### Nested PCR and 454-pyrosequencing of DNA samples

The *nifH* gene fragments were amplified from the cDNA samples acquired from ST.3, and from the genomic DNA samples acquired from all the Tokara Strait Stations (Table 2), following the nested polymerase chain reaction (PCR) protocol [5]. The nested PCR reaction was done in triplicate with the Platinum Taq DNA polymerase PCR system (Invitrogen, Thermo Fisher Scientific Corp.) in a volume of 12.5 μl containing 1 × rxn PCR buffer containing 4 mM MgCl_2_, 400 μM dNTPs, 1 μM primers (*nifH* 3 and *nifH* 4 for the first round, and *nifH* 1 and *nifH* 2 for the second round) [27], 1 unit Platinum Taq polymerase and 1 μL of total genomic DNA. Each round of the nested PCR was performed with 30 cycles of 94°C (1 minutes), 57°C (1 minutes) and 72°C (1 minutes). After the second round of the nested PCR, 1 μL of the PCR products was used to run 10 cycles of PCR with sample-specific multiplex identifiers (MIDs) and adaptor-attached primers [28]. The PCR conditions were identical to those of the nested PCR. The duplicates of the PCR products were mixed and then gel purified with a Quick Gel Purification kit (Invitrogen, Thermo Fisher Scientific Corp.). Negative controls, in which no template DNA was added, were also prepared for all the nested PCR of the genomic DNA samples. For the cDNA samples, the DNase-treated RNA samples were also added to the negative controls. According to the results of the DNA gel electrophoresis, no visible DNA bands were observed in the negative control lanes. For 454-pyrosequencing, MID-adaptor labeled *nifH* gene amplicons of different samples were mixed with the same concentration for constructing an amplicon library, according to the Rapid Library preparation protocol (Roche, 454 Life Sciences Corp., Branford, CT, USA). The DNA library attached beads were then loaded into a picotiter plate and sequenced with a GS Junior System (Roche, 454 Life Sciences Corp.).

**Table 2:** Environmental parameters of the various sampling locations.

Sample	Temp. (°C)	Salinity	NH_4_^+^ (nmol/L)	NO_2_^-^ (nmol/L)	PO_4_^3-^ (nmol/L)	SiO_2_ (μmol/L)	NO_3_^-^ (nmol/L)	*Chl a* (μg/L)	N/P
ST.1-S	24.15	34.51	45	55	19	2.23	57	0.37	7.66
ST.1-C	24.18	34.51	82	52	21.5	2.28	101	0.37	10.88
ST.3-S	24.34	34.42	72	130	36	2.30	64	0.55	7.47
ST.3-C	24.29	34.39	115	117	48	2.28	6	0.50	5.01
ST.4-S	23.30	34.23	75	341	70	2.67	178	0.58	8.54
ST.4-C	23.01	34.18	86	439	98	3.13	415	0.58	9.60
ST.5-S	22.40	34.14	105	302	98	3.38	409	1.07	8.36
ST.5-C	22.32	34.15	142	321	85	3.42	491	1.13	11.21
ST.6-S	21.74	34.29	31	156	117	3.39	580	0.95	6.58
ST.6-C	21.08	34.42	81	629	212	3.82	1370	1.33	9.83

### Quality control and analysis of sequences

The quality control of raw sequence data was conducted with the open source software package, Mothur [29]. Low quality sequences (with an average quality score < 25), short sequences (< 300 bases in length), ambiguous base containing sequences, homopolymer-containing sequences (i.e., homoploymers > 8 bases), and chimeric sequences were removed. Also, the multiplex identifier (MID) sequences were trimmed. The remaining sequences were de-noised with a sigma value of 0.01 to reduce the effect of PCR bias, after which they were aligned with reference sequences from the online *nifH* gene database of Ribosomal Database Project [30]. Based on 97% DNA similarity clustering of the high quality sequences, the operational taxonomic units (OTUs), representative sequences for each OTU, rarefaction curves, Good’s coverage indices, Shannon diversity indices and Chao richness estimators, were generated or calculated with Mothur [29]. The similarity among the samples was determined by clustering the samples with Braycurtis calculator using the Unweighted Pair Group Method with the Arithmetic Mean (UPGMA) algorithm in Mothur. The OTUs containing ≧ 20 sequences individually, were selected for the subsequent phylogenetic analysis.

In order to identify the OTUs, representative sequences were firstly translated into amino acid (aa) sequences using the FRAMEBOT online pipeline [31]. The aa sequences were used to search the protein sequences database on NCBI via the protein BLAST (Blastp) webpage [32]. The OTU representative sequences and the selected reference sequences from the NCBI protein sequences database were then aligned with ClustalW in MEGA 6.0 [33]. They were then used to construct a maximum likelihood (ML) phylogenetic tree based on the Poisson model in MEGA 6.0 [33]. The phylogenetic tree was further edited using iTOL [34].

Because new sublineages of UCYN-A have recently been discovered, and different sublineages can only be distinguished by their DNA sequences [14], higher resolution phylogenetic information regarding the cyanobacteria-like OTUs is needed. The DNA sequences of UCYN-A-like OTUs and of different sublineages of UCYN-A [14, 24] were aligned based on the respective amino acid translation using TranslatorX [35]. Then, the DNA alignment was used to construct a ML phylogenetic tree based on the Kimura 2-parameter model. Moreover, a similar phylogenetic tree was also constructed with the other cyanobacteria-like OTUs and their corresponding reference sequences. Based on these phylogenetic trees, the OTUs were grouped into different specific genera or clades. The relative abundances of the different genera or clades in different samples were calculated. All the data of 454-pyrosequencing of *nifH* amplicons obtained in this study have been deposited in the National Center for Biotechnology Information (NCBI) Sequence Read Archive (SRA) under the BioProject ID PRJNA356489.

### Statistical analysis

The correlations between the hydrographic variables were calculated with the Pearson test using Excel 2010. In order to reveal the relationships between the distribution of different cyanobacterial phylotypes and the environmental variables, the relative abundances of the cyanobacterial phylotypes in each sample were analyzed with detrended correspondence analysis (DCA) using Canoco 4.5 [36]. The environmental variables (Table 2) and the relative abundances of the cyanobacterial phylotypes were square-root transformed. Based on the DCA results, the longest length of gradient value among the 4 axes was between 3 and 4. Therefore, a redundancy analysis (RDA) was used to analyze the relationships among the relative abundances of cyanobacterial phylotypes and environmental variables in Canoco 4.5 [36]. The environmental variables with significant relationships (*p*-value < 0.05) were selected for explaining the diazotroph community variances, which were assessed in permutation tests with 499 unrestricted Monte Carlo permutations.

### Data analysis of an unpublished dataset collected in upstream of the Kuroshio Current

In the nucleotide database of the NCBI, there is a dataset of a clone library-based study conducted in the upstream of the KC, called “Seasonal variation in cyanobacterial diazotroph phylotypes in the northern South China Sea and the upstream Kuroshio”, which contained 294 sequences and has never been published in any literatures. The *nifH* sequences of species collected at a station in KC (~ 21.55°N, 122.10°E) during the spring, summer and winter, were analyzed using the same methods as the data of this study. The *nifH* sequences of different seasons were analyzed separately, and OTUs were calculated with Mothur [28], based on 97% similarity of the DNA sequences. Because there were approximately 5 sequences per sample in this dataset, which is insufficient to reconstruct the diazotroph community structure. Therefore, the objective of analyzing this dataset is to find the common OTUs of the KC in the Tokara Strait and the upstream of KC, instead of comparing the diazotroph community structures of the two regions.

## Results

### Hydrography and nutrients

**Fig 2:**
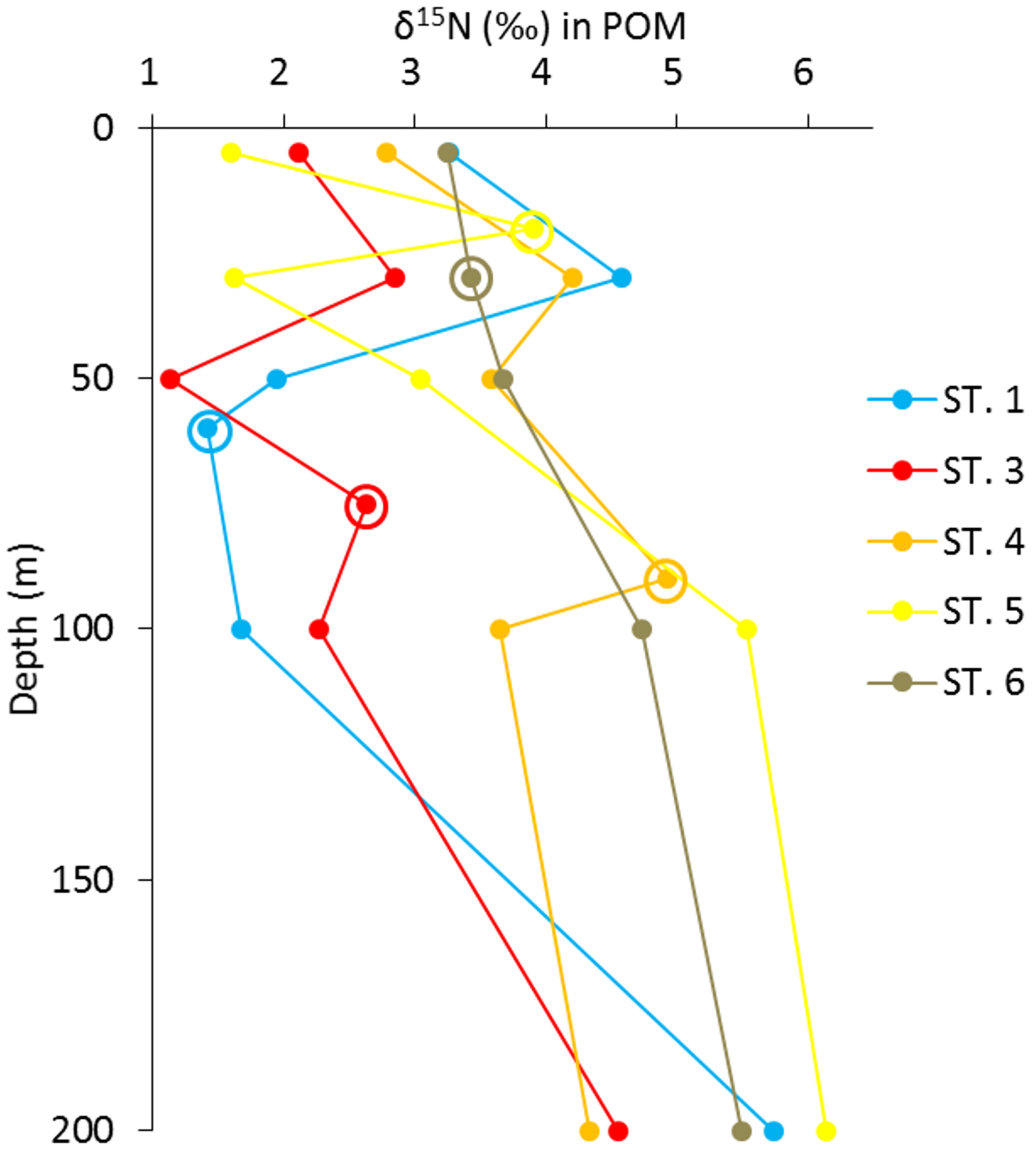
The stable isotope ratio of nitrogen (δ15N) in particular organic matter (POM) at the different stations. The samples collected from the deep chlorophyll maximum (DCM) layer are indicated with circles.

According to the surface current data from the ship-mounted ADCP and the path of KC from the “Quick Bulletin of Ocean Conditions” provided by the Japan Coast Guards (S1 Fig), ST.3 was located in the core of KC, and ST.4 was located in the boundary of KC. Along the ST.1 to ST.6 transect, apparent gradients of temperature and nutrients were observed. The highest sea surface temperature occurred at ST.3, which was at the core (flow axis) of the KC, and the lowest temperature was observed at ST.6 (Fig 1, Table 2). Concentrations of macro-nutrients (NO_2_^-^, NO_3_^-^, SiO_2_ and PO_4_^3-^) increased from ST.1 and ST.3 to ST.6. The physico-chemical parameters suggest the possible influence of nutrient input from Kuroshima Island and Kyushu Island to the studied transect. A higher salinity was also observed at ST.6, suggesting that the nutrients might also be replenished with the islands induced upwelling. The *Chl a* concentration showed positive and strong correlation with the nutrients (S1 Table). The lowest nitrate concentration (10 nmol/L) and N/P ratio (5.01) were detected at ST.3. This N/P ratio is clearly lower than the Redfield ratio (i.e., N/P ratio = 16) [37]. In addition, at ST.3, the stable isotope ratios of nitrogen (δ^15^N) in particulate organic matter (POM) were generally lower than that at the other stations (Fig 2). With regards to the DCM samples, the δ^15^N values at ST.1 (1.42‰) and ST.3 (2.62‰) were lower than those at ST.4 -6 (3.42‰ - 4.92‰).

### Diazotroph community diversity and phylogeny along the studied transect

**Fig 3:**
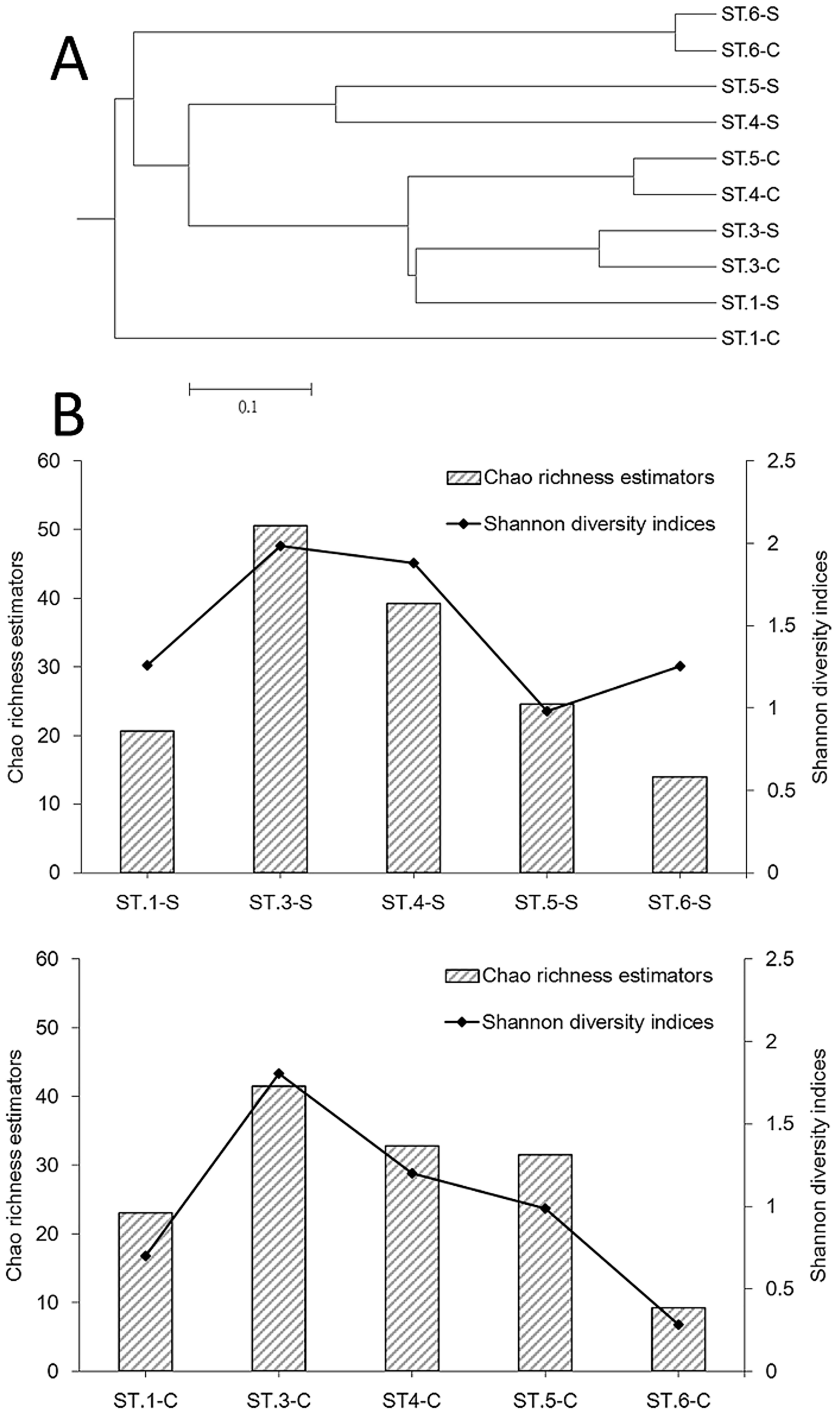
(A). A UPGMA dendrogram showing the relationship between samples, at 0.03 cutoff. (B). Shannon diversity indices and Chao richness estimators of the diazotroph community in the samples, at 0.03 cutoff.

In total, 41,284 high quality sequences were included in this study. For all the samples, the Good’s coverage values were between 0.996–0.999 (Table 1) and the rarefaction curves nearly plateaued (S2 Fig), which means that the sequencing depth of this study is enough to represent the community structure of the diazotrophs. For the similarity of the diazotroph community among the samples (Fig 3A), ST.3-S and ST.3-C clustered to each other. The diazotroph community structure at ST.3 also clustered with that of the nearby stations (i.e., ST.1, 4 and 5), with different degrees of similarity. Both the diversity and richness indices reached peaks in the ST.3-S and ST.3-C, and decreased from ST.3 to the nearby stations (Fig 3B).

**Fig 4:**
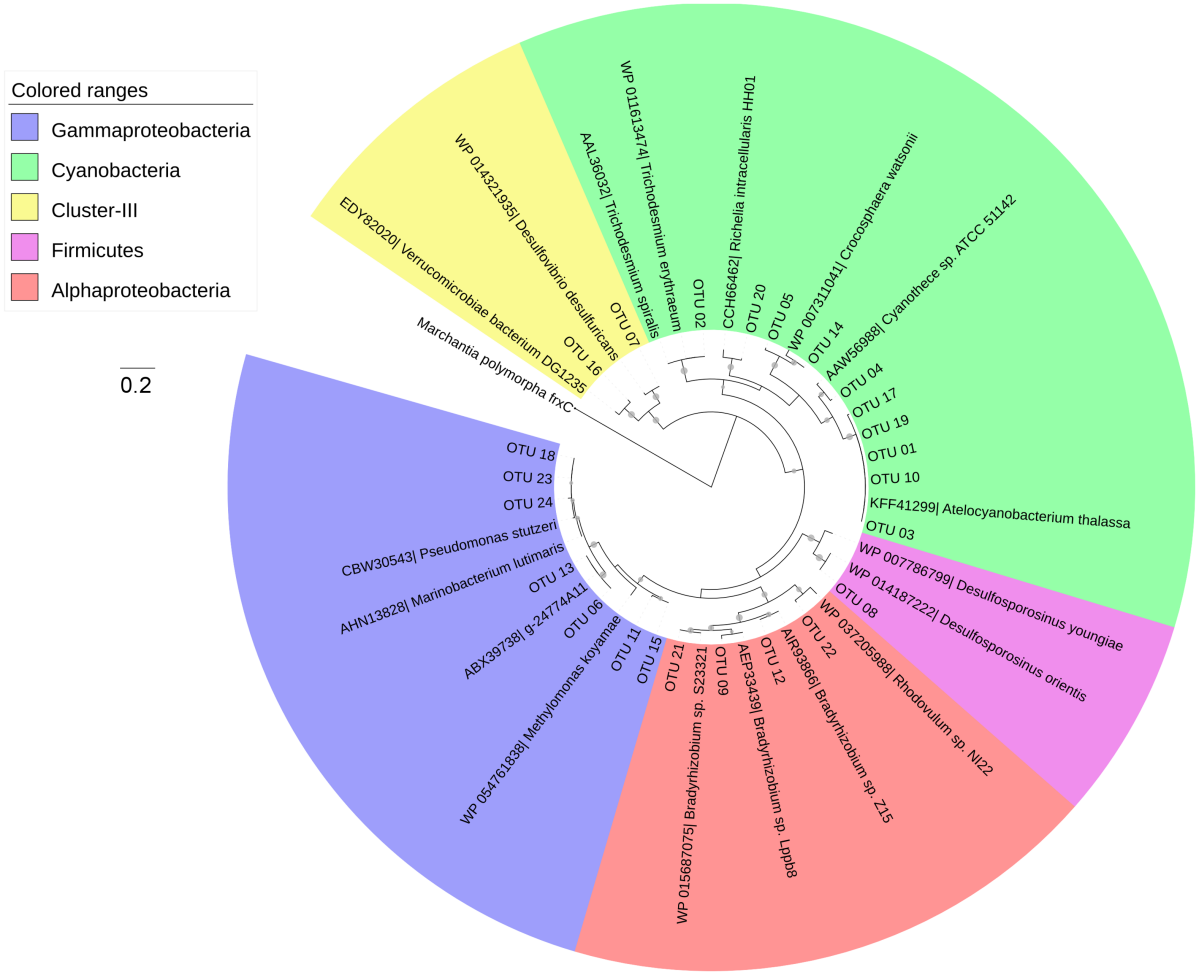
A maximum likelihood phylogenetic tree constructed with amino acid sequences of the *nifH* gene. Representative sequences of the top 24 OTUs and the affiliated reference sequences were used to construct this tree. The accession numbers of each reference sequence are shown in front of the sequence description. Bootstrap resampling was performed 1,000 times; and the values that are higher than 50% are labeled with grey circular symbols on the branches.

After removal of the OTUs containing < 20 sequences, there were 24 OTUs (accounting for 96.7% of the original 41,284 sequences) remaining for further analysis. Nearly half of the OTUs fell into the cyanobacterial cluster (i.e., 10 / 24 OTUs), followed by *Gammaproteobacteria* (7 / 24 OTUs), *Alphaproteobacteria* (4 / 24 OTUs), Firmicutes (1 / 24), and cluster-III anaerobes (2 / 24 OTUs) (Fig 4).

**Fig 5:**
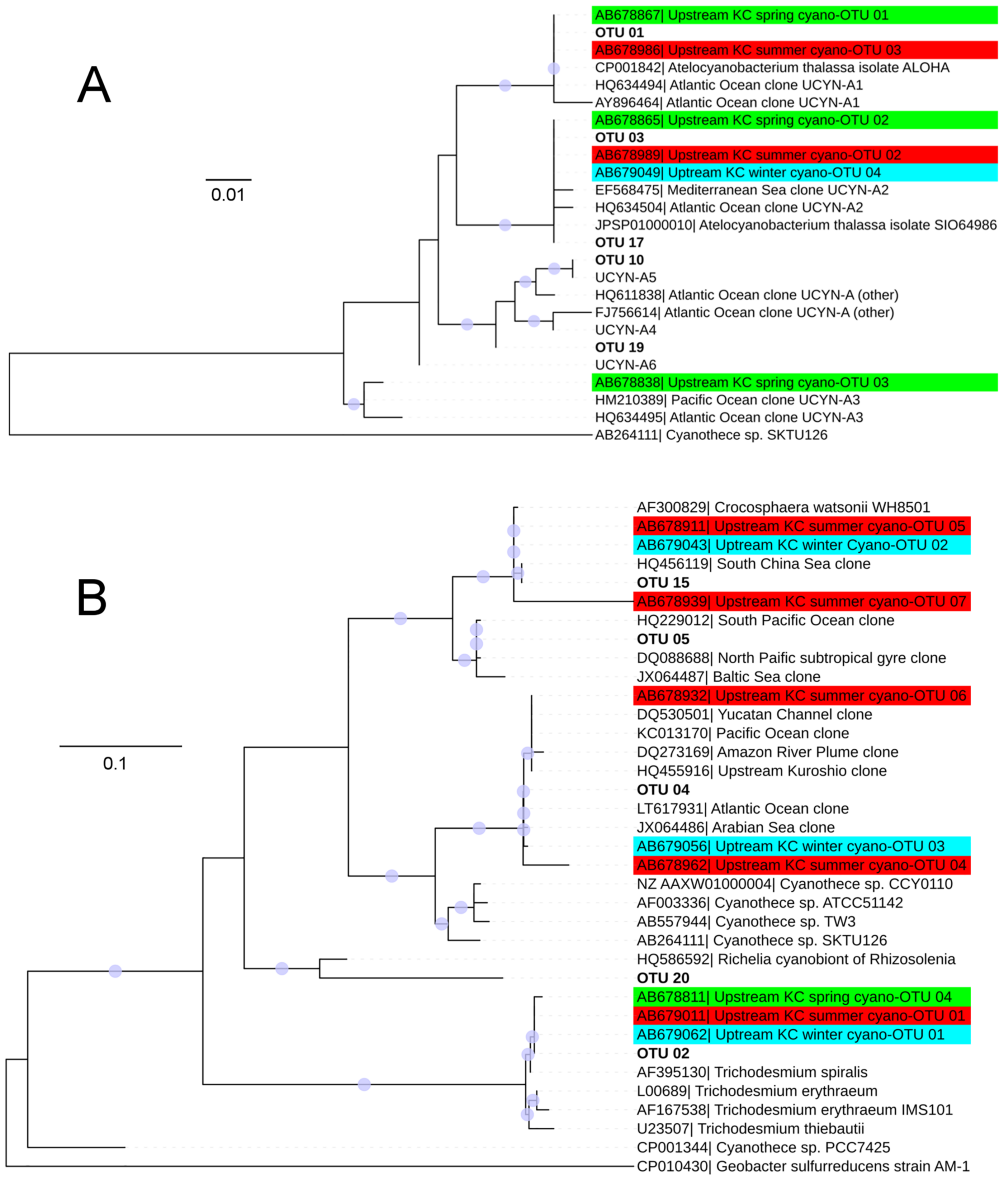
Maximum likelihood phylogenetic trees constructed with *nifH* DNA sequences of (A) UCYN-A sublineages and (B) the other cyanobacterial diazotrophic phylotypes. The corresponding accession numbers are displayed in front of the description of the reference sequences. The sequences labeled with “UCYN-A4”, “UCYN-A5” and “UCYN-A6” represent the OTUs of corresponding phylotypes in the NGS based study of Turk-Kubo et al [24], which do not have accession numbers. Bootstrap resampling was performed 1,000 times; and the values that are higher than 50% are labeled with blue circles on the branches. The OTUs identified in the Tokara Strait are in bold, and the OTUs identified from the unpublished data in the upstream region of the KC, are labeled with “upstream KC” and highlighted in blue, green, or red for winter, spring, and summer respectively.

Within the group of cyanobacteria, a number of different clades could be distinguished via DNA-based ML phylogenetic trees (Fig 5). Among UCYN-A, UCYN-A1, UCYN-A2 and UCYN-A (other) were detected (Fig 5A), based on the classification of the previous definition [14]. For UCYN-B, an OTU (OTU 5) which has 94% similarity in DNA with *Crocosphaera watsonii* (accession no.: AF 300829) was discovered. The result of phylogenetic analysis showed that the OTU 5 belongs to the cluster of UCYN-B; and there are two clades within this cluster (Fig 5b). We named OTU 5 containing clade UCYN-B2, and we renamed the clade (OTU 15 containing) that closely affiliates to *Crocosphaera watsonii* (previously named UCYN-B) UCYN-B1 (Fig 5B). For UCYN-C, OTU 04 showed a 92% genetic (DNA) similarity with that of *Cyanothece sp*. TW3 isolated from the upstream KC (Fig 5B). By searching the NCBI nucleotide database (Blastn), the *Cyanothece*-affiliated environmental clones showed high similarity with OTU 04 (i.e., 99–97% similarity in DNA), which in turn showed ≦ 92% similarity in DNA with all the *nifH* sequences of the *Cyanothece* species (Fig 5B). The OTU 02 found in the KC was affiliated to *Trichodesmium spiralis*.

### Diazotroph community and activity in the core of KC

**Fig 6:**
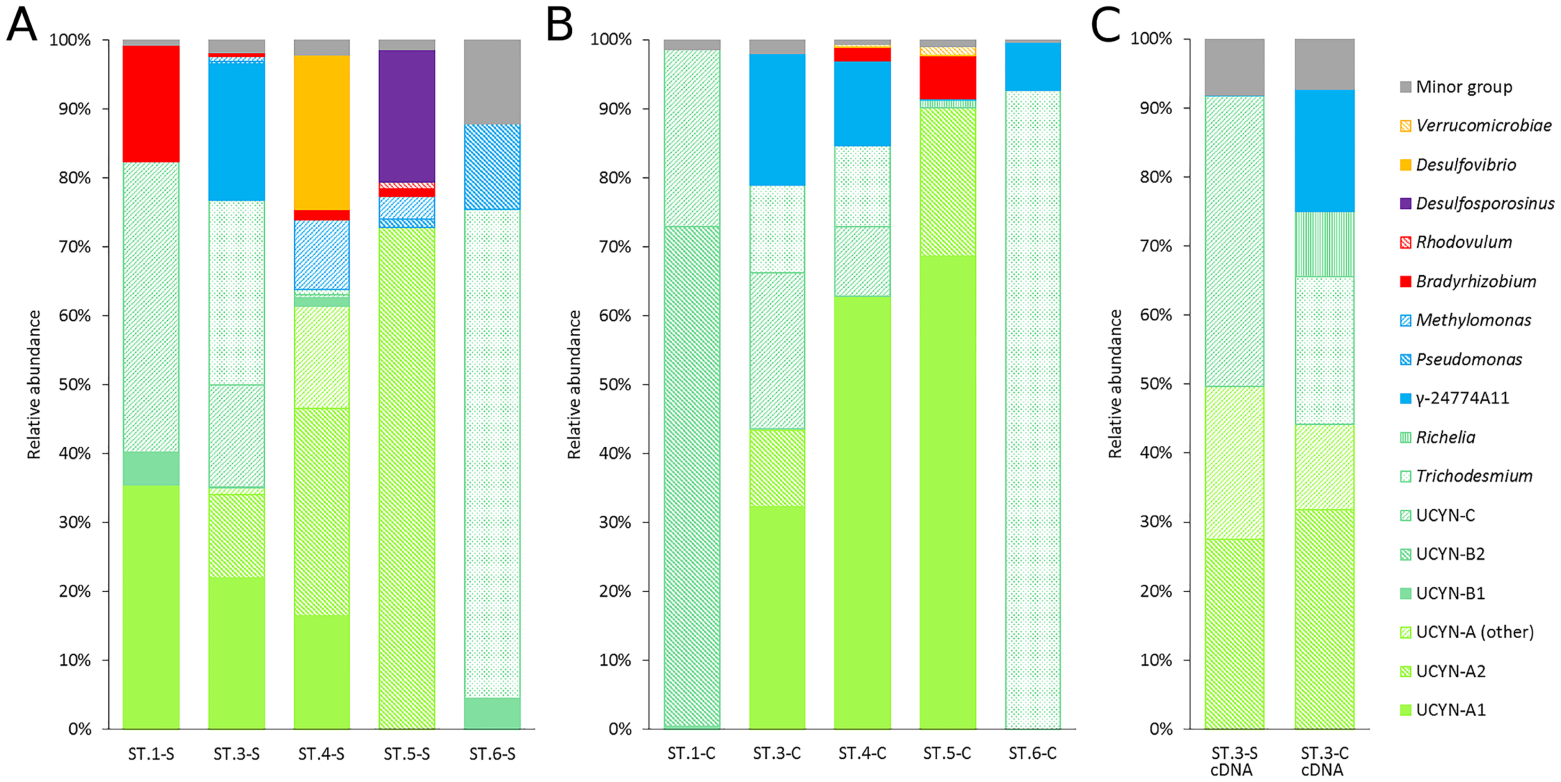
The relative abundance of the various diazotroph clades. The cyanobacteria are labeled in shades of green, the *Gammaproteobacteria* are in blue, the *Alphaproteobacteria* are in red, the cluster-III diazotrophs are in yellow, and the Firmicutes are in purple. The abundance of the diazotroph clades are shown in (A) the surface and (B) DCM waters of all the stations, as well as (C) for two cDNA samples collected in the surface and DCM layers of ST.3.

Apart from the cluster-III diazotrophs and UCYN-B2, almost all the phylotypes of diazotroph discovered in this study were detected at ST.3, which was the core of the KC (Fig 6). The unicellular diazotrophs contributed 71% and 85% of the diazotroph community at ST.3-S and ST.3-C, respectively. UCYN-A was the most dominant diazotroph at ST. 3, contributing 35% and 44% of the communities at ST.3-S and ST.3-C, respectively. *Trichodesmium* contributed 27% and 13% of the diazotroph communities at ST.3-S and ST.3-C, respectively, while the remaining ~40% of the communities were contributed equally by UCYN-C and γ-24774A11. Within the UCYN-A group, UCYN-A1 was the most abundant sublineage (making up 22%– 33% of the whole community), whereas UCYN-A2 also contributed a significant proportion of the group (i.e., ~12% of the diazotroph community). A small amount of UCYN-A (other) was also detected at ST.3 (Fig 6). The OTUs of UCYN-A1, UCYN-A2, UCYN-B1, UCYN-C and *Trichodesmium* sp. detected in both the upstream and Tokara Strait regions showed >99% similarity in DNA (Fig 5).

For the cDNA results, UCYN-A1 was not detected among the top 24 OTUs (Fig 6). However, both UCYN-A2 and UCYN-A (other) contributed to significant portions of the transcript. The UCYN-A2 group contributed 27% and 32% of the *nifH* transcripts at ST.3-S and ST.3-C, respectively, while UCYN-A (other) contributed 22% and 12% of the *nifH* transcripts at ST.3-S and ST.3-C, respectively. Significant contributions of UCYN-C (44% at ST.3-S) and *Trichodesmium* (26% at ST.3-C) to the *nifH* transcripts were also detected. In addition, γ-24774A11 contributed 18% of the *nifH* transcripts in the DCM water at ST.3 (Fig 6).

### Spatial variations of the diazotroph community along the Tokara Strait

Along the Tokara Strait, UCYN-A was in most cases the most dominant group, contributing 36% to 90% of the total diazotroph community. However, at ST.1-C, ST.6-S and ST.6-C, either UCYN-B2 or *Trichodesmium* dominated (Fig 6). Within the UCYN-A group, the relative abundance of UCYN-A1 was in general higher (accounting for 16–69% in different samples) than that of UCYN-A2 and UCYN-A (other). The relative abundance of UCYN-A2 increased from ST.3 (12%) to ST.5 (73%) in the surface water, and its relative abundance was lower in the DCM water. UCYN-A (other) was detected at ST.3-S, ST.3-C and ST.4-S, and it reached the highest relative abundance (15%) at ST.4-S. The second most dominant group along the transect was *Trichodesmium*, which was detected at ST.3, 4 and 6. However, while there was a predominance of *Trichodesmium* (71–93% of the community) at ST.6 (which was close to the islands of Kyushu and Kuroshima), its relative abundance at both ST.3 and ST.4 was lower than that of UCYN-A.

The UCYN-C group was only detected at ST.1 - ST.4, and the relative abundance of this group decreased in both the surface (from 42% to 0.4%) and DCM (from 26% to 10%) waters from ST.1 to ST.4 (Fig 6). The UCYN-B1 group as well as the diatom-associated *Richelia* were detected as rare species in some samples. The *Gammaproteobacteria* diazotrophs (including γ-24774A11) and *Bradyrhizobium* reached ~20% of the whole community in some of the oligotrophic samples (i.e., ST.1-S, ST.3-S and ST.3-C), whereas they remained just a small proportion (i.e., < 10%) of the community in the remaining samples. The cluster-III diazotrophs were only found at ST.4 and ST.5.

### Environmental variables and cyanobacterial diazotrophs

**Fig 7:**
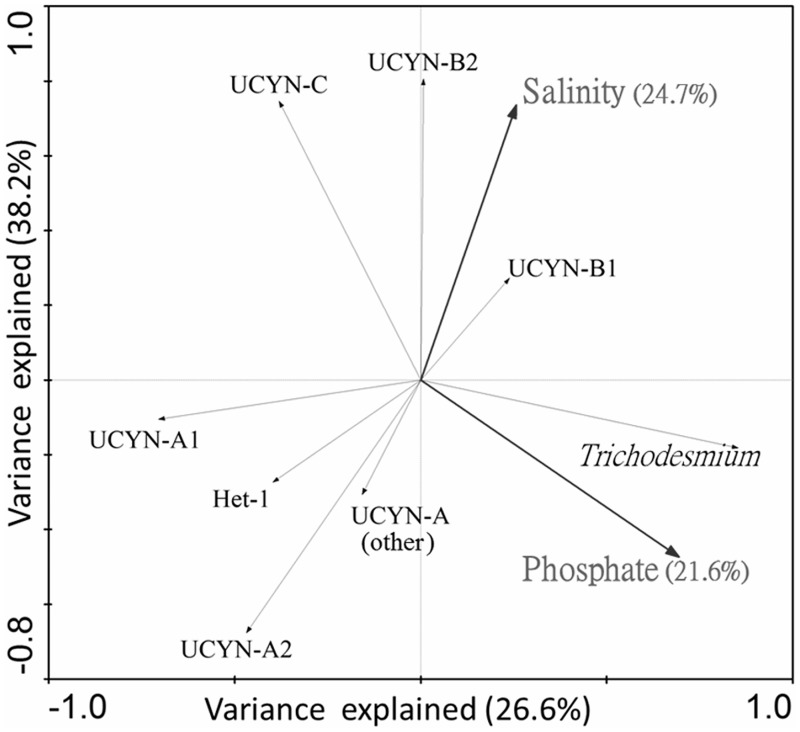
Correlation biplot based on a redundancy analysis (RDA), depicting the relationship between the relative abundances of the cyanobacterial clades and the environmental factors. The percentage of variance explained by the environmental variables are shown in the brackets.

Among all the environmental parameters measured (Table 2), the salinity and phosphate levels showed significant correlations with the relative abundance of the cyanobacterial diazotrophs, and together they explain 46.3% of the variances (Fig 7). UCYN-A2 and UCYN-A (other) showed negative correlations with the salinity; UCYN-C showed a positive correlation with the salinity and a negative correlation with phosphate levels; and *Trichodesmium* showed a positive correlation with the phosphate levels.

## Discussion

### Diazotrophy and diverse unicellular diazotrophs in the KC

With the lowest nitrate concentration and N/P ratio among all sampled stations (Table 2), ST.3 showed the highest diversity of diazotrophs, which suggests that diazotrophs might take advantage of the N-limited condition at the core of the KC [21]. It has been reported that the δ^15^N value of oceanic nitrate is ~ +5‰ [38], while that of fixed nitrogen is close to 0‰ [39, 40]. Therefore, a lower δ^15^N value in the POM might indicate that nitrogen fixation is an important contributor of the new production [41]. Thus, the lower δ^15^N values in POM at ST.3 (Fig 2) indicate higher activity of nitrogen fixation in the core of the KC. Although the ^15^N signature is just an indirect indicator of nitrogen fixation, our interpretation agrees with the previous reports that nitrogen fixation is an important nitrogen source of new production in KC [18, 19].

Although UCYN-A1 and UCYN-B1 were reported as the major diazotrophs in KC in a previous qPCR-based study [42], our data showed that the summation of UCYN-A2, UCYN-C and γ-24774A11 comprised over half of the diazotroph community in this area. Since diverse unicellular diazotrophs and *Trichodesmium* were detected in KC at both DNA and cDNA samples, the contribution of different phylotypes to the potentially strong nitrogen fixation in the core of KC is worthy for future studies.

### Sublineages of UCYN-A in studied area and the coastal preference of UCYN-A2

The group UCYN-A was the most predominant group of the diazotrophs identified in the core of the KC (i.e., at ST.3) and the adjacent waters (Fig 6), where the UCYN-A1, UCYN-A2 and UCYN-A (other) were all detected. According to previous reports, UCYN-A1 can be detected in regions with a temperature range of 15°C– 30°C [43–45], and the highest abundance is detected at 24°C [8]. The sea surface temperature from ST.1 to ST.5 ranged from 22.32°C to 24.34°C, which might explain why the UCYN-A group was predominant along the Tokara Strait. By comparing with the reference sequences of UCYN-A sublineages of the most recent study [24], OTU 10 (UCYN-A other) affiliates to the newly defined UCYN-A5 (Fig 5A). Our findings agree with previous reports suggesting that some of the UCYN-A sublineages might have an overlapping distribution [14, 24, 25]. The differences in the relative abundance of the various UCYN-A sublineages in our cDNA results (Fig 6) might be caused by different diel patterns of *nifH* gene expression. Although UCYN-A2 and UCYN-A1 were reported to have similar diel patterns of *nifH* gene expression [14], different *nifH* gene expression patterns of spatially separated UCYN-A1 have also been reported [46]. Therefore, the diel patterns of *nifH* gene expression of the different UCYN-A sublineages are still waiting to be clarified. Nevertheless, the significant contribution of UCYN-A2 and UCYN-A (other) at the level of cDNA (Fig 6), suggests that these 2 sublineages should also be considered as functionally important diazotrophs in the KC.

It was suggested that UCYN-A2 may prefer coastal conditions [14, 47]. However, later studies based on qPCR data and distributional pattern of ribosomal RNA gene sequences of UCYN-A and the host of UCYN-A suggested that both UCYN-A1 and UCYN-A2 are globally distributed [48, 49]. A more recent study reported that the qPCR primers targeting UCYN-A2 were not specific, which may be able to hybridize with the *nifH* gene of UCYN-A3 [25]. Besides that, deep sequencing data of samples from different regions of the ocean showed that UCYN-A3 is more abundant than UCYN-A2 in oceanic samples, while high relative abundance of UCYN-A2 was only detected in the coastally-influenced samples [24]. In our study, the relative abundance of UCYN-A2 increased gradually in the surface water from offshore station to the near-shore station (Fig 6), which implies the preference and the importance of UCYN-A2 in coastal water.

Moreover, the relative abundance of UCYN-A2 and UCYN-A (other) were higher in the surface water (Fig 6) than in the DCM, which suggests that they prefer the hydrographic conditions in surface water. It has been reported that the relative abundance of UCYN-A3 was higher in mid depths in the Northeast Pacific Ocean [24], however, UCYN-A3 was absent in our samples. Therefore, the factors that affect the global distribution of UCYN-A sublineages are still waiting to be unraveled.

### Unexpected high relative abundance of UCYN-C in the KC and offshore stations

Besides UCYN-A, UCYN-C was also the key unicellular cyanobacterial diazotroph in the KC and the offshore stations, and it was also detected in the upstream region of the KC in both summer and winter (Fig 5B). It is unexpected, because UCYN-C was not reported being a major diazotroph phylotype in the Pacific Ocean and the adjacent South China Sea [43, 44, 50, 51]. Based on the distributional pattern of UCYN-C (Fig 6) and the RDA result (Fig 7), it is suggested that UCYN-C prefers low phosphate and high salinity oceanic waters in the KC and western North Pacific. Together with the recent result of a phosphate enrichment mesocosm experiment that UCYN-C was more competitive when dissolved inorganic phosphate (DIP) was depleted [52], it provides further evidence on the relationship between phosphate and UCYN-C.

Moreover, a recent study has reported the evidence that UCYN-C derived nitrogen was directly ingested and assimilated by zooplanktons [53], while those derived from diatom diazotroph association and *Trichodesmium* was not detected in the same study. Although UCYN-C was less well-studied than other cyanobacterial diazotrophs in previous studies [54], a recent study has shown that low concentration of UCYN-C was detected throughout the surface waters of tropical North Atlantic basins [49]. Considering that it could bloom and has the highest growth rate among the reported data of other diazotrophs when the favored conditions are fulfilled [52], the importance of UCYN-C in the marine biogeochemical cycling and microbial food web may be overlooked in the past. Because of the high relative abundance of UCYN-C detected in this study, the West Pacific Ocean and KC is an ideal region to study the importance of UCYN-C.

*Cyanothece* sp. TW3, which was isolated from the upstream region of the KC near Taiwan, was proposed as the only one culture of UCYN-C [10]. However, like other species of *Cyanothece*, the *nifH* sequence of *Cyanothece* sp. TW3 is phylogenetically distant (i.e., with 92% similarity in DNA) from the cluster of the previously reported UCYN-C-affiliated environmental clones (Fig 5B). Therefore, the phylogeny of UCYN-C and the representativeness of the current culture of UCYN-C (*Cyanothece* sp. TW3) are worthy to be studied in future.

### Genetic diversity of UCYN-B sublineages

The genetic diversity of UCYN-B was reported to be low, in which different strains of *Crocosphaera watsonii* and environmental clones of UCYN-B from the global oceans shared highly conserved *nifH* and 16S rRNA gene (> 98% similarity in DNA) [55]. On the top of the view that the *nifH* sequences and other gene makers of UCYN-B were highly conserved [55, 56], two phenotypes (sub-populations) of UCYN-B were identified with different cell size, which have differences in genome, physiological characteristics and distributional pattern [9, 56, 57]. Therefore, the identification of the two sublineages of UCYN-B in this study provides new insight to the micro-diversity of UCYN-B. The UCYN-B1 has > 98% similarity in DNA with different strains of *Crocosphaera Watsonii* (including the previously mentioned two phenotypes), which was widely detected in the global oceans [55]. For the UCYN-B2, the affiliated sequences (≧ 97% similarity in DNA) were also detected in different regions, including the North and South Pacific Ocean [7, 22, 58], North Atlantic Ocean [48] and the Baltic Sea [31] (Fig 5b). Moreover, the predominance of UCYN-B2 at ST.1-C (Fig 6) implies its potential significance in the western North Pacific Ocean, which is worthy of further studies. With further works on deep sequencing of *nifH* gene fragment, more sublineages of UCYN-B might be discovered, similar to the case of UCYN-A [24, 25].

### Predominance of *Trichodesmium* near the islands

A predominance of *Trichodesmium* was observed at ST.6, a station close to the islands of Kuroshima and Kyushu, where the highest concentration of macro-nutrients indicates the presences of terrestrial input (Table 2) [59]. On top of that, the obvious elevation of salinity and decrease in temperature at ST.6 (Table 2) can be an indicator of upwelled subsurface water [60]. The nitrate rich upwelled water was thought to inhibit nitrogen fixation, however, enhanced nitrogen fixation was observed in the equatorial upwelling region of Atlantic Ocean where N/P ratio was low [61]. It was suggested that the high concentration of phosphate and iron in the upwelled water could be advantageous to diazotrophs, as long as the N/P ratio is low [61]. The N/P ratios at ST. 6 ranged from 6.58 to 9.83 (Table 2), which are lower than the value of the Redfield’s ratio (i.e., N/P = 16) [37], thus was suitable for the proliferation of *Trichodesmium*. Indeed, a number of studies have reported that *Trichodesmium* can bloom or be abundant in the waters close to islands [17, 62, 63], and the input of iron and phosphate from the islands were suggested to be the reasons of this phenomenon. Our data suggested that island induced upwelling of subsurface water, which is rich in P and Fe relative to N, could be another reason for the high abundance of *Trichodesmium* at this station.

### Highly heterogeneous diazotroph community and the role of KC in transportation of diazotrophs

With the passage of the KC from the East China Sea to the Pacific Ocean and the interaction of KC and islands, the Tokara Strait displays a steep environmental gradients (Table 2). Concurrently, highly variable diazotroph communities were also observed along the transect. Such variations can be explained by the differential responses of UCYN-A2, UCYN-C and *Trichodesmium* to the environmental gradients, as discussed in the previous parts. On the top of that, the remarkable changes of the relative abundances of UCYN-A2 (12–73%) and UCYN-C (42% - 0.4%) within a small geographic scale (29°N—31°N 129°E—130°E) suggested that diazotrophs are highly sensitive to changing environmental conditions. In an earlier study conducted in the Northeast Pacific Ocean, high-resolution sampling has revealed that the abundance of diazotrophs can change with nearly 3 orders of magnitude within less than 2 days and 30 km, which was correlated to the changing environmental conditions associated with mesoscale eddies [64]. Our results reiterate the importance to carry out high-resolution sampling for understanding the high variability of diazotroph community in the highly dynamic oceans [64].

The majority of cyanobacterial OTUs that were detected in this study have also been detected in the upstream region of the KC near Taiwan (Fig 5). This suggests that the diazotrophs are transported by the KC from the southeastern waters of Taiwan to the Tokara Strait, and it was also proposed that phytoplankton might be transported in the KC from tropical to temperate regions [65]. On the other hand, UCYN-A3 was detected with the clone library analysis in the upstream KC, but it was not detected with the deep sequencing of this study (Fig 5A), highlighting the dynamics of the diazotroph community structure in different regions along the path of KC. Therefore, studies aiming to understand the dynamics of diazotrophs in this fast moving water mass over a large geographic scale is also needed for better understanding of the factors and mechanisms that control the distribution of the diazotrophs and hence nitrogen fixation in the global oceans.

## Summary

We described the comprehensive structure of the diazotroph community at both the DNA and cDNA levels in the KC and its adjacent waters. Highly heterogeneous diazotroph composition and distribution observed in this relatively small study area was mainly due to the different responses of diazotrophic phylotypes in response to the steep environmental gradient resulted from the interaction of KC and the adjacent islands. Moreover, our findings highlight the importance to study the dynamics of diazotroph composition and activities in both mesoscale features with strong hydrographic gradients and large geographic region, such as the whole KC from upstream to extension, in order to better understand the factors that control the distribution of different diazotrophs and their nitrogen fixation in global oceans. In addition, our results about the phylogeny of UCYN-A, UCYN-B and UCYN-C provide new information for understanding the sublineages of the unicellular cyanobacteria diazotrophs.

## Supporting information

S1 Fig The path of KC and the surface current data during the cruise. (PDF)

S2 Fig Rarefaction curve of the observed OTUs of the samples at 0.03 cutoff. (TIFF)

S1 Table Results of the Pearson test of paired environmental variables. Pearson correlation coefficients that are significant (at *p* < 0.05, *n* = 10), are labeled with asterisks.
(DOCX)
