# Supplementary figures and images for: Highly heterogeneous diazotroph communities in the Kuroshio Current and the Tokara Strait, Japan
Source: PLoS One. 2017 Oct 23;12(10):e0186875. doi: 10.1371/journal.pone.0186875 (PMC5653367; doi:10.1371/journal.pone.0186875)

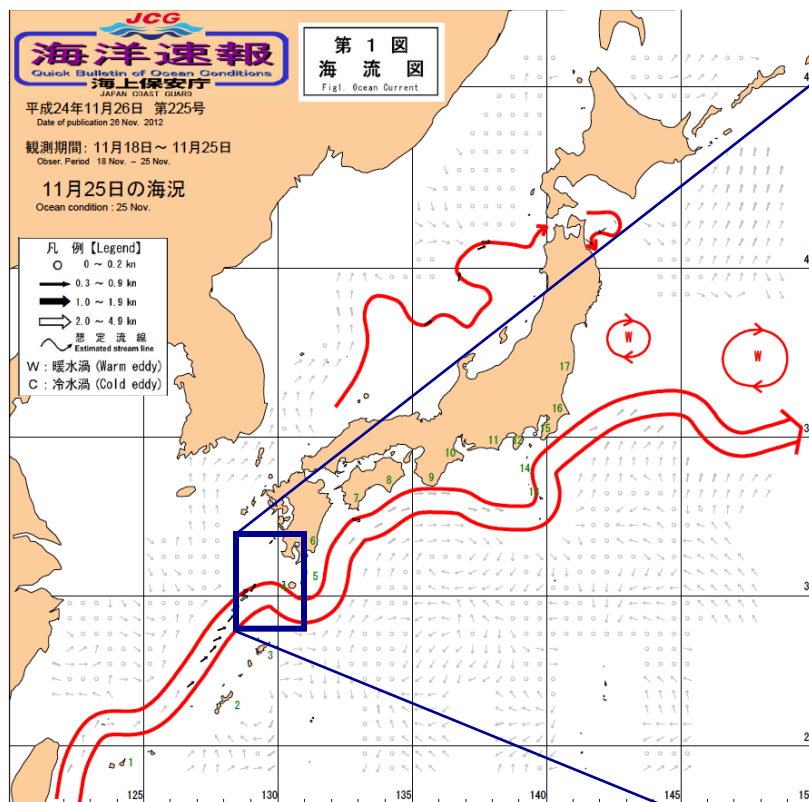

淡青丸 KT-12-31 Track Chart('12 11.16 ~ 11.23)(Scale: 1 / 1500000)

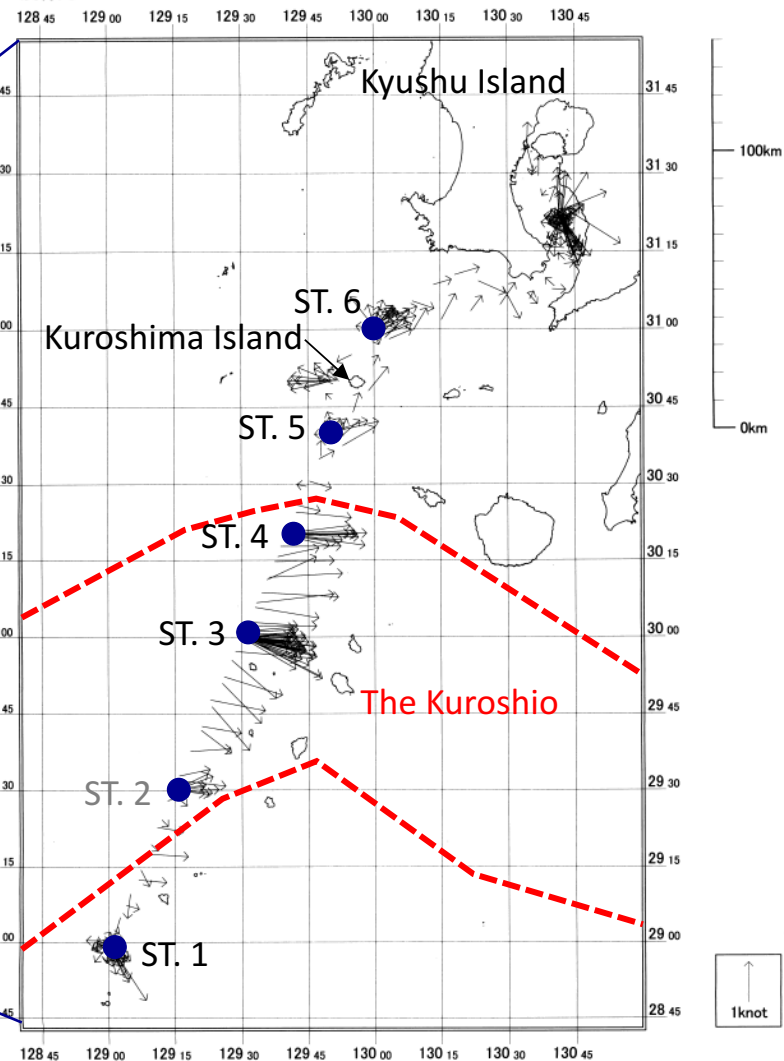

Supplement: S1 Fig — (PDF) [file pone.0186875.s001.pdf]

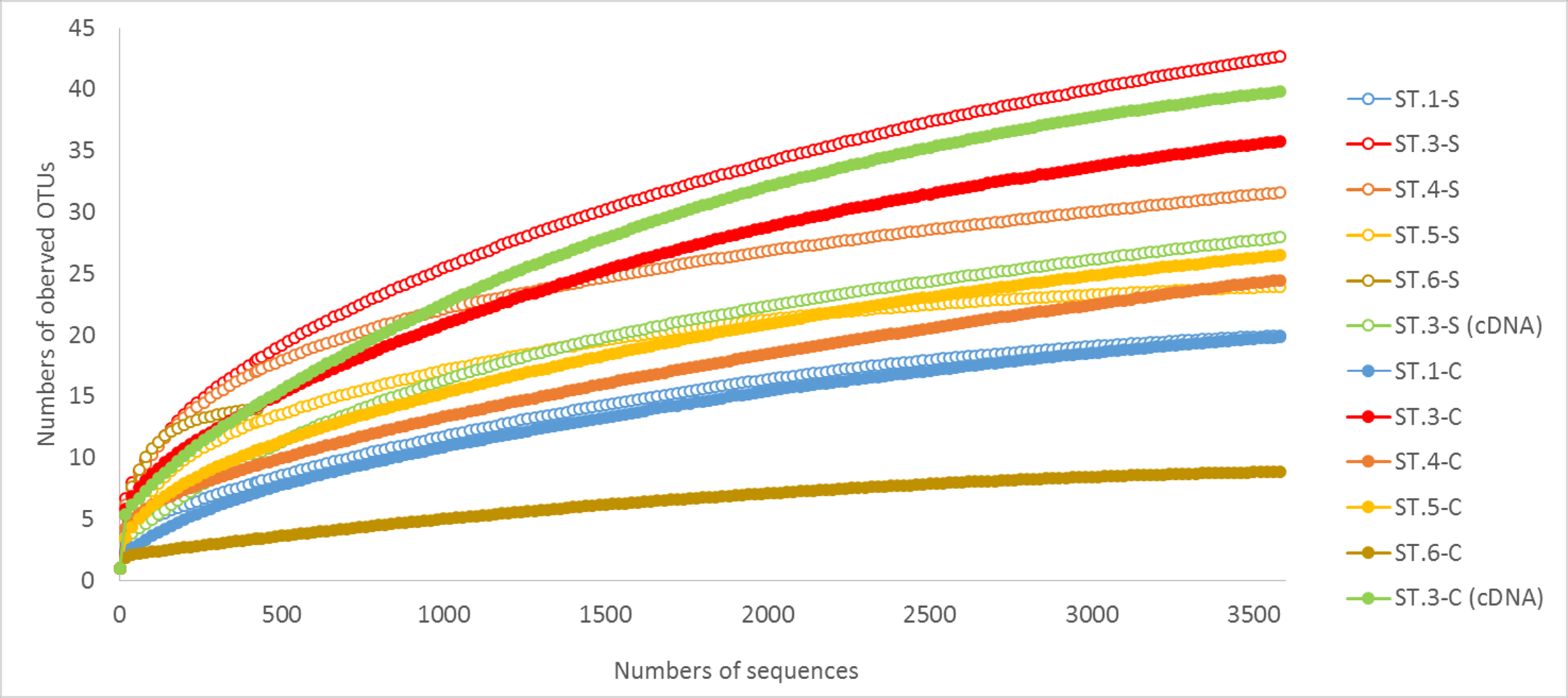

Supplement: S2 Fig — (TIFF) [file pone.0186875.s002.tiff]
