# Supplementary material for: Highly heterogeneous diazotroph communities in the Kuroshio Current and the Tokara Strait, Japan
Source: PLoS One. 2017 Oct 23;12(10):e0186875. doi: 10.1371/journal.pone.0186875 (PMC5653367; doi:10.1371/journal.pone.0186875)
Supplement: S1 Table — Pearson correlation coefficients that are significant (at p < 0.05, n = 10), are labeled with asterisks. (DOCX) [file pone.0186875.s003.docx]

**S1 Table. Results of the Pearson test of paired environmental variables.** Pearson correlation coefficients that are significant (at *p* < 0.05, *n*=10), are labeled with asterisks.

| Temp. | 1.00 |  |  |  |  |  |  |  |  |
| --- | --- | --- | --- | --- | --- | --- | --- | --- | --- |
| Salinity | 0.46 | 1.00 |  |  |  |  |  |  |  |
| NH_4_^+^ | -0.05 | -0.52 | 1.00 |  |  |  |  |  |  |
| NO_2_^-^ | *-0.74 | -0.46 | 0.31 | 1.00 |  |  |  |  |  |
| PO_4_^3-^ | *-0.93 | -0.33 | 0.07 | *0.87 | 1.00 |  |  |  |  |
| SiO_2_ | *-0.98 | -0.58 | 0.21 | *0.79 | *0.91 | 1.00 |  |  |  |
| NO_3_^-^ | *-0.91 | -0.17 | 0.03 | *0.82 | *0.97 | *0.88 | 1.00 |  |  |
| *Chl a* | *-0.92 | -0.48 | 0.30 | *0.70 | *0.87 | *0.93 | *0.85 | 1.00 |  |
| N/P | -0.29 | -0.18 | 0.36 | 0.41 | 0.20 | 0.36 | 0.35 | 0.28 | 1.00 |
|  | Temp. | Salinity | NH_4_^+^ | NO_2_^-^ | PO_4_^3-^ | SiO_2_ | NO_3_^-^ | *Chl a* | N/P |
